# Definition of a sectioning plane and place for a section containing hoped-for regions using a spare counterpart specimen

**DOI:** 10.1038/s41598-022-17380-z

**Published:** 2022-08-03

**Authors:** Zhongmin Li, Goetz Muench, Clara Wenhart, Silvia Goebel, Andreas Reimann

**Affiliations:** Advancecor GmbH, Lochhamerstr. 29 A, 82152 Martinsried, Germany

**Keywords:** Biological techniques, Cell biology, Structural biology, Anatomy, Medical research

## Abstract

Histological examination of targets in regions of interest in histological sections is one of the most frequently used tools in biomedical research. However, it is a technical challenge to secure a multitarget section for inspection of the structure’s mutual relationship of targets or a longitudinally filamentous- or tubular-formed tissue section for visitation of the overall morphological features. We present a method with a specified cutting plane and place, allowing researchers to cut directly at the multitarget centers accurately and quickly. The method is proven to be reliable with high accuracy and reproducibility and a low coefficient of variation, testing on repeat experiments of three target’s position-known models. With this method, we successfully yielded single sections containing whole intraorbital optical nerves, three aortic valves, or whole thoracic tracheas in their central positions. The adjoined custom-made tools used in the study, such as various tissue-specific formulated calibrated trimming and embedding guides, an organ-shaped cavity plaster mold, and a two-time embedding technique for optimal and identical trimming or embedding, also bear great potential to become a common supplemental tool for traditional histology and may contribute to the reduction of the labor, and the number of animals needed.

## Introduction

Morphological and imaging analysis of targets in the regions of interest is essential for studying cellular morphology and phenotypes in situ and the diagnosis of local lesions. If there is just one target in the region of interest, the requirement for visualization and analysis in one section is relatively easy to meet by histological serial sectioning. This approach uses continuous inspection with a microscope or stereomicroscope, where serial sectioning is carefully carried out until the desired plane and section position is achieved. Such an approach would require the excellent craftsmanship of the operator. However, this will become a much more cumbersome and technically challenging process if a longitudinal section of filamentous- or tubular-formed organs (e.g., nerves, muscles, vessels, tracheas) is required for the visitation of overall morphological features or if a section containing more than one target is needed for the inspection of the structure’s mutual relationship of targets. There are no existing methods for alignment of a sectioning direction along a longitudinally cutting plane of the whole filamentous- or tubular-formed organs in tissue blocks or a sectioning plane to hit two or more targets in one stroke.

The standard sectioning planes—sagittal, transversal, and coronal, which are mainly used to cut and describe the tissue structures and the target location of one region of interest in anatomy, histology, and imaging medicine, are not properly sufficient to be applied for sectioning and description of the general morphological features of a whole filamentous- or tubular-formed organ and the structure’s mutual relationship of targets in the region of interest, such as correlation, relative localization, extrusion, intrusion, or connection in between.

For example, we require an image of one section in orbits, which shows a whole intraorbital optic nerve lineament. In such a case, applications of any one of three standard sectioning planes in orbital serial sections do not meet the requirement. The serial sections only exhibit one part of the tissue of interest in one section. Incorrect sectioning direction or cutting-forward position/distance would lead to an unsuccessful experiment with no possibility of recovery.

Defining the desired cutting planar to hit all the target centers in regions of interest concurrently can be particularly difficult. Difficulties arise due to limitations in visualizing the positions of the targets contained within the tissue embedding block. Difficulties also arise in specifying the cutting plane and position that strokes the target centers in one section.

Micro-computed tomography (micro-CT)^1^, nano-computed tomography (nano-CT)^1,2^, and ultrasound- or magnetic resonance imaging (MRI)- guided histological sectioning planes have been reported recently^3–5^. However, those studies focus only on one target of the region of interest and the methods work under the condition that the region is large enough to be compatible with the resolution of the imaging machines and that the special tissues to be examined (e.g., bone or teeth) produce an obvious density contrast between the targets and the surrounding tissues. In addition, access to imaging equipment is not available in every laboratory, and thus, a method that has widespread applicability will be greatly appreciated.

**Figure 1:**
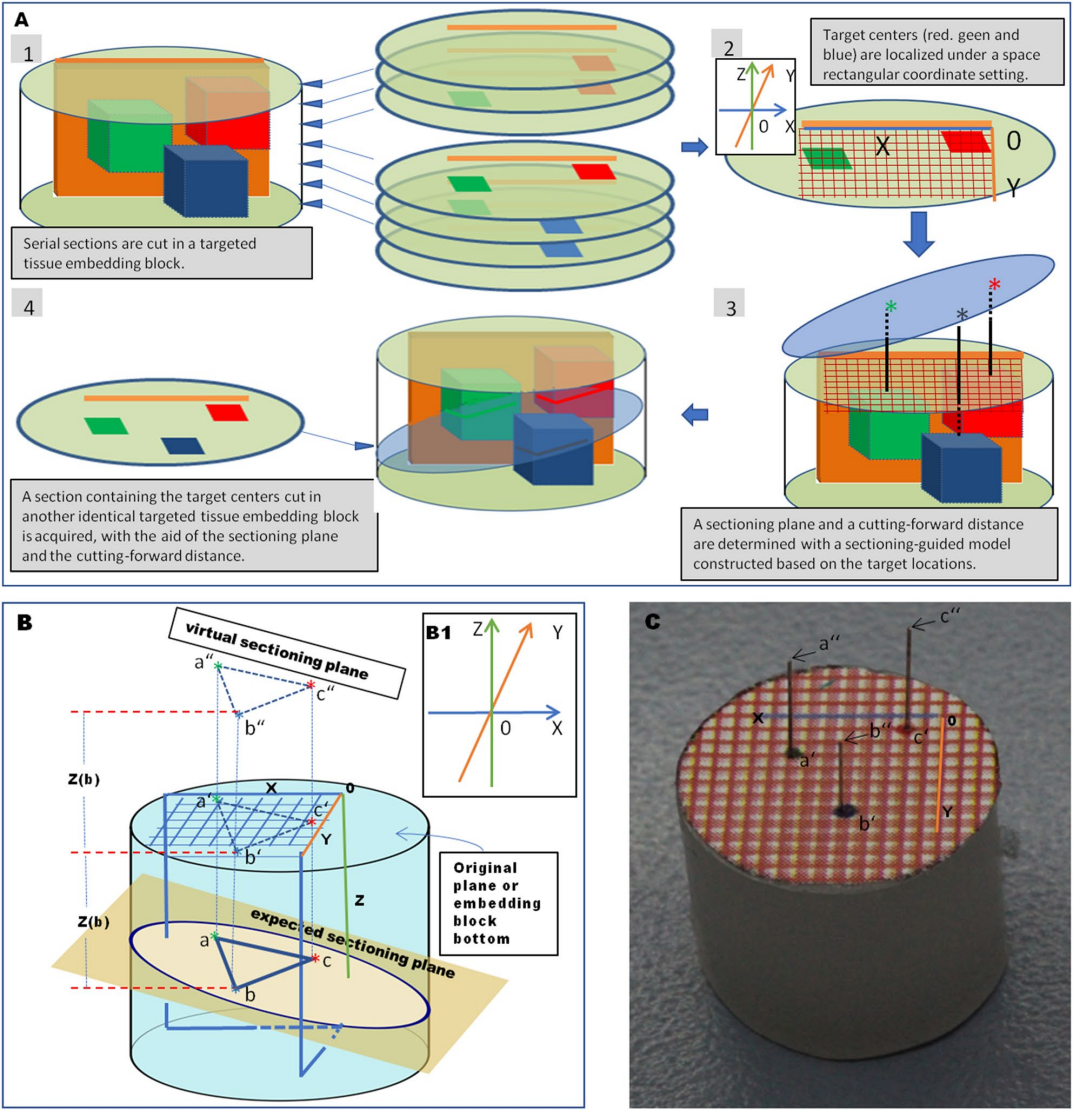
Experimental flow chart and fundamentals for determination of a sectioning plane and cutting-forward distance. (**A**). An experimental flow chart of 4 steps. (**B**). A three-targeted model (embedding block) is created. The 3D central positions of the three targets are a (− X_a,_ − Y_a,_ − Z_a_), b (− X_b,_ − Y_b,_ − Z_b_), and c (− X_c,_ − Y_c,_ − Z_c_). A sectioning plane (light yellow) that hits those three target centers (a, b, and c) is required (expected sectioning plane). A suppositional cutting plane—a‘-b‘-c‘ is parallel to the expected sectioning plane—a-b-c and the virtual sectioning plane—a“− b“− c“ with an interval of Z(b) (in the supposition of the lowest value of − Z_b_ among three Z values— − Z_a_, − Z_b_ and − Z_c_). The central spatial positions in the virtual plane are [− X_a,_ − Y_a,_ (2 × Z_b _− Z_a_)] for a“, [− X_b,_ − Y_b,_ Z_b_] for b“ and [− X_c,_ − Y_c,_ (2 × Z_b _− Z_c_)] for c“. (**C**). A sectioning-guided model is made of an identical paraffin block to (**B**) in size and with the virtual sectioning plane (a”− b”− c”) formed by the tips of three needles based on the corresponding central positions of the targets.

In this report, we describe a method of defining a sectioning plane and position to secure a section containing a whole filamentous- or tubular-formed organ outline or two or more hoped-for target centers. For this, four separate steps are needed (see Fig. 1A).

In the first step, we used a specimen embedding block (data-out block) of one animal to localize the tissue targets in a virtual space rectangular coordinate setting formed by a positioning plate (X-axis, see Methods) inset in the block. Second, we prepared a sectioning-guided model (see Methods) according to the target central spatial positions (X, Y, Z). Third, the sectioning plane or cutting angle was adjusted according to the sectioning-guided model, and the cutting-forward distance/place required to hit the target centers was calculated. Fourth, using the corrected sectioning plane and cutting-forward distance, the histological section containing central aspects of the targets is finally produced in another identical sample embedding block (data-in block) from a similarly sized and weighting animal.

The diagram in Fig. 1B is intended to help understand the principle behind the method. A virtual space rectangular coordinate setting is formed by a positioning plate (or reference, see Methods) inset in the embedding block (the blue line of a positioning plate border functions as an X-axis, the orange perpendicular virtual line through one end of the plate or an original point on the horizontal plane serves as a Y-axis, and the green line in cutting-forward direction is regarded as a Z-axis. The three-dimensional (3D) central positions (X, Y, Z) of the targets—a (− X_a,_ − Y_a,_ − Z_a_), b (− X_b,_ − Y_b,_ − Z_b_), and c (− X_c,_ − Y_c,_ − Z_c_)—are determined in the data-out block (Fig. 1B), and an a-b-c plane is defined (expected sectioning plane in Fig. 1B). The plane rises vertically by the value of Z (b) along the Z-axis to the suppositional a’− b’− c’ plane and goes further up by the same amount of height to the virtual sectioning plane of a”− b”− c”. The planes of a-b-c, a’− b’− c’ and a”− b”− c” are parallel to each other, and the distances between the planes are equal. Note that the Z value of b is supposed to be a minimal value (the largest absolute value) among those of the three targets in the Z-axis of the present setup. A corresponding sectioning-guided model indicated in Fig. 1C is constructed. To uphold the target space positions, we used a length of fine needles for the Z-axis and the positions of the tips for the target centers, and three needle tips form a plane (a”− b”− c”) in Fig. 1C, which is equivalent to the virtual sectioning plane in Fig. 1B. The tip spatial positions are [− X_a,_ − Y_a,_ (2 × Z_b_ − Z_a_)] for a“, [− X_b,_ − Y_b,_ Z_b_] for b“ and [− X_c,_ − Y_c,_ (2 × Z_b_ − Z_c_)] for c“. Adjust and fix the cutting plane or angle so that the blade hits all three tips of needles concurrently at a cutting stroke. Use the corrected sectioning plane and start to cut sections in the data-in identical embedding block of a similar-sized animal (data-in animal) until to hit b’ marked on the embedding block bottom. The specimen cutting-forward distances are from the virtual sectioning plane to the position to hit b' by the blade. With the defined sectioning plane, the hoped-for section is obtained at the position of the cutting-forward distance for further investigations.

For validation of the method, we utilized custom-made three-targeted models, in which the three target positions (X, Y, Z) were known. The results showed high accuracy and repeatability and a low coefficient of variation on the position and cutting-forward distance detected with respect to the foreknown values, which confirms the method to be reliable.

Using the technique, we successfully displayed the whole intraorbital optic nerve lineament in one section from the right-side orbit of the data-in mouse. In the same way, sections of three aortic valves in their central positions were produced in the heart. We also observed a central aspect of the whole thoracic trachea in one section of the thoracic segment. Repeatability testing on these samples further revealed that the method is reliable and robust.

## Results

### Accuracy and repeatability

**Figure 2:**
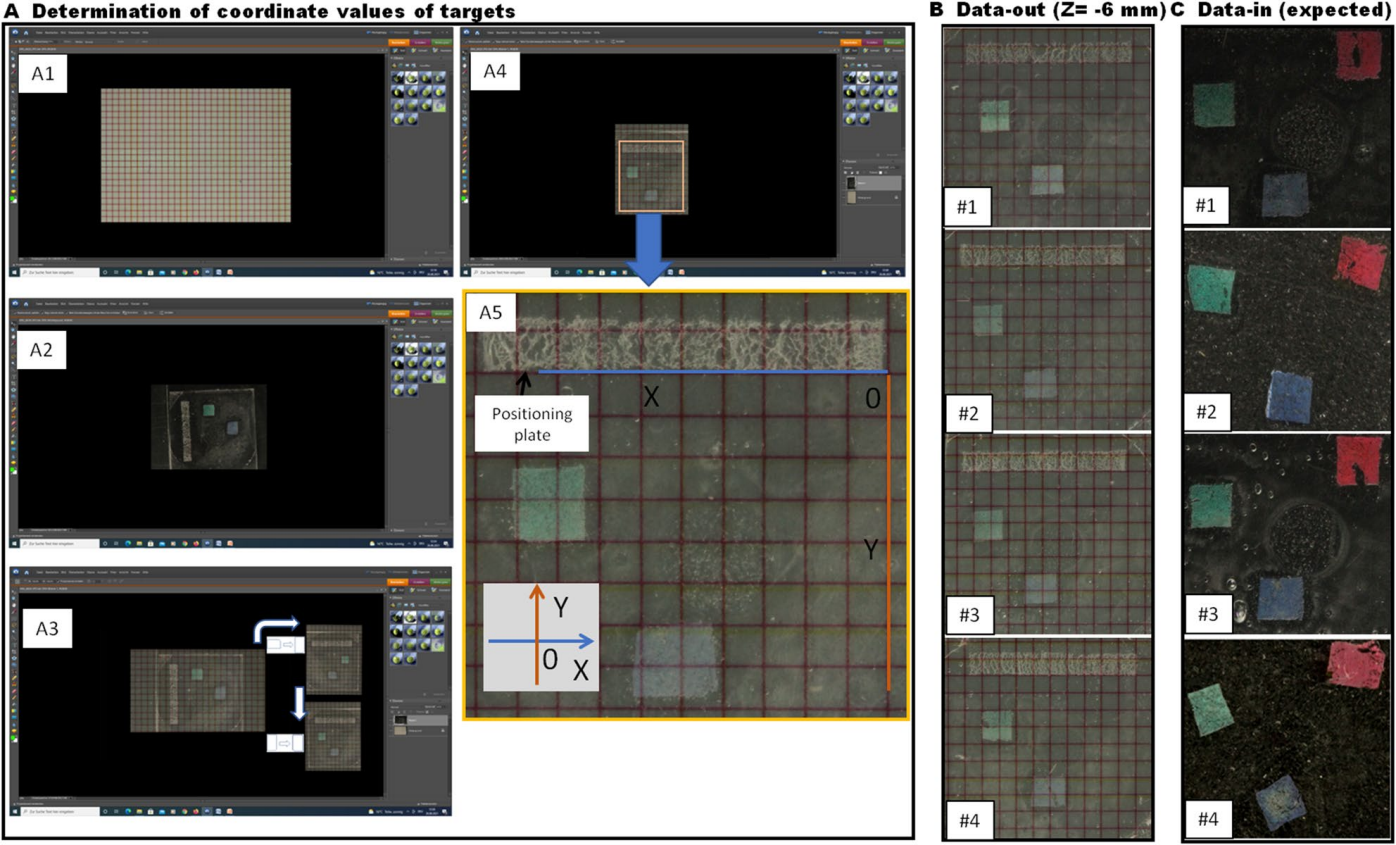
Localization of targets in data-out three-targeted models and the sections cut in paired data-in models. Secondary to serial sections cut in the data-out models, the coordinate values of target positions (green and blue) are determined in reference to red grid squares and a positioning plate (**A**). (**B**). Representative sections cut in the distal part (Z = − 6 mm) of the three-targeted data-out models (N = 4) display similar locations of the targets. With the sectioning-guided planes, the sections [Data-in (expected)] are collected at the expected places (corresponding predetermined cutting-forward places) in the data-in paired three-targeted models (N = 4), which contain all the targets (**C**). A5 is a high magnification of the local in A4.

**Figure 3:**
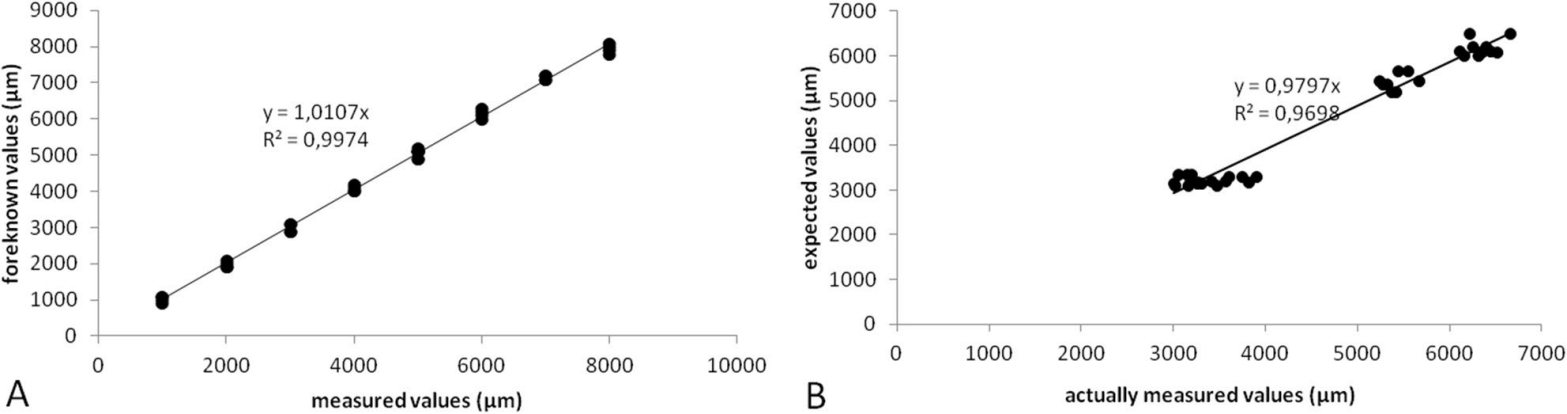
Accuracy tests on the target central coordinate value of foreknown and measured with the method in three-targeted models (**A**), and on specimen cutting-forward distance/place of actual and expected for targets in data-in tissue blocks (**B**). Comparison of the values between foreknown and measured in (**A**) and of the values between expected and actually measured in (**B**) yield statistically significant correlation in both (**A**) and (**B**). [N = 36, Pearson’s r = 0.998699, p < 0.0001 of 2-tailed for (**A**); and N = 36, Pearson’s r = 0.985, *p* < 0.000193 of 2-tailed, for (**B**)].

To validate the protocol, we created eight identical three-targeted models (see Methods), in which the central positions of the three targets in the embedding blocks were known. Those models were randomly made into four pairs. One model in a pair served as a data-out model, and the other model served as a data-in model. The 3D coordinate values at the central positions (X, Y, Z) of the targets measured in the four data-out models were determined in four different periods (Table 1, see also Fig. 2A5/B). The results showed that there was a high similarity in each coordinate value between the foreknown and measured values or among the measured values of different rounds (Table 1). Multiple comparisons between the known and the measured or among the different measured rounds with Tukey resulted in no statistical significance (N = 45, for all, *p* = 0.9999857–1). The overall coefficient of variation for the four runs was 0.03134, ranging from 0.093 to 0.008. The similar locations of the targets observed in the distal part (Z = − 6 mm) of the three-targeted data-out models (N = 4) are demonstrated in Fig. 2B. For the accuracy of the technique, we pooled the values of the three target positions in X, Y and Z, measured in the data-out models. Comparison of the foreknown values with the values measured yielded a statistically significant correlation (Fig. 3A), and there was a high closeness between the measured and the foreknown values (N = 36, Pearson’s r = 0.998699, *p* < 0.0001 of 2-tailed).

**Table 1:** Foreknown central positions of three targets measured in the data-out molds and the coefficients of variation (CV) from the measurements in four different periods.

Targets	Axes	Foreknown (μm)	Measured (μm)				
			First	Second	Third	Fourth	CV
Red	X	− 2000	− 1900.0	− 2100.0	− 2000.0	− 1900.0	0.04848
	Y	− 1000	− 900.0	− 1100.0	− 1000.0	− 1100.0	0.09341
	Z	− 4000	− 4100.0	− 4200.0	− 4000.0	− 4000.0	0.0235
Green	X	− 8000	− 7900.0	− 8100.0	− 8000.0	− 7800.0	0.01624
	Y	− 3000	− 2900.0	− 2900.0	− 3100.0	− 3100.0	0.03849
	Z	− 5000	− 5100.0	− 5200.0	− 5100.0	− 5100.0	0.00976
Blue	X	− 5000	− 4900.0	− 4900.0	− 5100.0	− 5100.0	0.02309
	Y	− 7000	− 7200.0	− 7100.0	− 7100.0	− 7200.0	0.00807
	Z	− 6000	− 6100.0	− 6300.0	− 6000.0	− 6200.0	0.02099
Total							0.03134

Multiple comparisons between foreknown and measured values or among different rounds measured, with Tukey HSD, result in no statistical significance (N = 45, for all, *p* = 0.9999857–1).

According to the target positions measured in the data-out models, the corresponding sectioning-guided models were constructed (refer to Methods), and the corresponding sectioning planes and expected cutting-forward distances (the values in brackets of Table 2) were determined. With the sectioning planes, sectioning was performed in the paired data-in models. To test the accuracy, the central positions (actual cutting-forward distances) where the sections were cut for each target were recorded. A high similarity in the cutting-forward distances between expected (predetermined from the sectioning-guided model) and actual paired data-in blocks was found (Table 2). The overall coefficient of variation for four runs on different days was rather small (0.068, for actual; and 0.0445, for expected), with an arrangement of 0.05–0.08 for actual measurements. Comparisons with Student paired T-tests resulted in no statistical significance (N = 12, *p* > 0.192 of 2-tailed). With the method, we successfully acquired the section at the place of the expected cutting-forward distance, which contains three targets in their central or proximate positions in all the repeat experiments (N = 4) of data-in models (Fig. 2C).

**Table 2:** Repeatability testing on specimen holder cutting-forward distances (µm) calculated from the sectioning-guided models (expected) and measured in paired data-in models (actual), and the coefficients of variation (CV) from four runs in different periods.

Targets	First	Second	Third	Fourth	CV actual	CV exp
Red	6050 [6100]	7050 [6700]	6680 [6118]	6150 [6380]	0.07228	0.0445
Green	6150 [6100]	7150 [6700]	6070 [6118]	6160 [6380]	0.08042	
Blue	6040 [6100]	6500 [6700]	6472 [6118]	6850 [6380]	0.05131	
Total					0.068	0.0445

Comparisons between the expected (in brackets) and actual values with Student’s paired T-tests produce no statistical significance (N = 12, *p* > 0.192 of 2-tailed). The numerals without brackets indicate the actual values measured in data-in models.

The results reflect a rather high accuracy and reproducible technique over time and confirm that the method is feasible and robust. To further validate the method, we extend the application to the tissue blocks.

### Application in tissue sectioning

There are many requirements in biomedicine and clinics for inspection of the whole targeted region outlines of filamentous- or tubular-formed tissue, or different targets with respect to a landmark in the central positions on one section. We selected some often-used organs for sectioning (1) to further validate the method and (2) to meet the requirements with the organ-specific detailed sectioning protocols. These organs represent differently sized levels from small (aortic root, 6 × 6 × 5 mm^3^) through the middle (orbit, 12 × 8 × 6 mm^3^) to large (thorax, 25 × 25 × 17 mm^3^).

#### Aortic root sectioning

The aortic root is in the position of three aortic valves, a place prone to developing atherosclerosis. The degree of atherosclerotic severity in the region of aortic valves is one of the important indexes for anti-atherosclerotic drug effectiveness^6–8^. Standardized sections through three-valve centers will be essential for the valid comparison and judgment of atherosclerotic severity. In addition, atherosclerosis induces aortic valve stenosis, which is a well-known valvular heart disease worldwide^9^. The popular surgical procedure and minimally invasive transcatheter aortic valve implantation are used to treat severe stenosis^10^. Before the surgical procedure, assessing aortic parameters with respect to the valve landmarks is required. The typical procedure used is time-consuming and complicated^11–14^. A highly precise section containing the three-valve centers, which will meet the requirements, calls for a divergent sectioning method from the existing ones.

#### Orbital sectioning

The most characteristic pathologic findings seen in Graves' ophthalmopathy are enlargement of the intra-ocular muscles, edema of the fatty and muscular orbital tissues, and more advanced pathological changes of infiltration of the tissue by plasma cells, lymphocytes, mast cells, macrophages, and fibrosis^15–22^. The results are mostly derived from locally isolated intraorbital tissues without an anatomical orientation or standard sectioning plane. This incurs a high risk of misinterpretation for examination of the pathological changes when a comparison is made for the effectiveness of treatments. Therefore, these studies require inspection of pathological changes with respect to the whole intraorbital optic nerve lineament present. The optic nerve is widely accepted to be one of the most important landmarks for the localization or measurement of local lesions in the orbits. Thus a standardized section with the whole intraorbital optic nerve lineament presented is required for valid comparison.

#### Thoracic sectioning

Toxicological studies on the effects of inhalable particles and fibers often include an assessment of histopathological alterations in the tracheas and lungs. Conventional pathological evaluations are usually performed on separate tissues after the dissection of a body^23,24^. This approach not only comprises a potential cutting position or direction bias of the tissues but also conveys the risk of mild structural alterations of those organs after isolation from the thoracic cavity. In addition, the trachea might undergo a distinct change in the geometry after dissection, since aortas in similar elastic tissues become much shorter in the separate state than in the physical or in vivo situation^25,26^. Thus, it is important to make diameter measurements in situ of whole intact sections to reconstruct the morphometries of the thoracic trachea. The most accurate and simple way of obtaining unbiased information is employing a whole thoracic trachea longitudinally sectioning, which goes through the whole thoracic trachea in its central position. In addition, the section obtained will be visualized for whole structural alternations in the thoracic trachea, and the precise site-specific morphological changes that respond to outside stimulations can be further determined. However, it is difficult to obtain such a section with an existing method reported.

**Figure 4:**
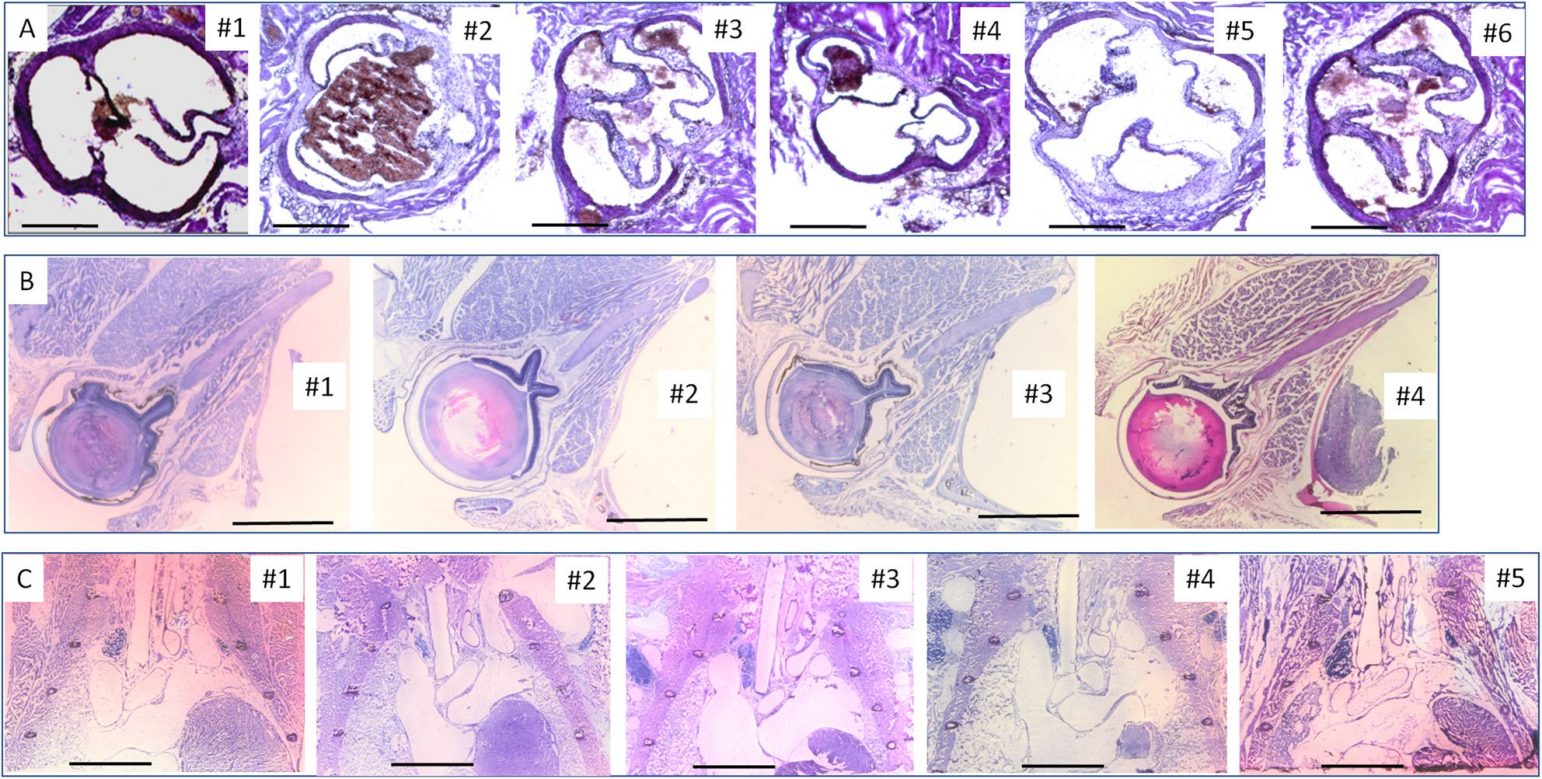
Images of the sections cut at expected cutting-forward distance/places in the paired data-in blocks. The sections cut in data-in paired blocks, containing the whole targets or the lineaments in their central positions are secured, as shown in (**A**, **B**, and **C**). (**A**). The sections cut at aortic roots of the hearts in 6 rounds of experiments. Three complete valves are shown in each section. —, indicates 0.5 mm. (**B**). The sections cut in orbits in 4 rounds of experiments. The intraorbital optic nerve between the duct and the connection with the eyeball is shown in each section. —, indicates 2.0 mm. (**C**). The sections cut in thoraxes in 5 rounds of experiments. The thoracic trachea is shown in each section. —, indicates 4.0 mm.

To acquire a section containing the targets or those with respect to a landmark, the specimens were made into pairs with similarity (Table 3) in age and body weight of sex and went through an identical trimming and embedding for each pair. One specimen in the pair serves as the data-out specimen and the other data-in specimen. After serial sectioning, staining, and photographing, the central positions (X, Y, Z) of the targets of the data-out blocks were determined (see Methods and Fig. 2A), with reference to the positioning plate inset. The central positions of the targets measured are listed in Tabs S1, S2, and S3 from the data-out blocks of orbits, hearts, and thoraxes, respectively (see also Fig. 4). The results showed that there was a high similarity in the coordinate values of position for each targeted organ among the 4–6 rounds measured. Multiple comparisons among the different rounds measured for each kind of sample, with Tukey HSD, led to no statistical significance (N = 24–54, for all, *p* = 0.970857–1). The overall coefficients of variation in the repeated measurements on different days were very low, ranging from 0.0299 to 0.0541.

**Table 3:** Body or related organ weight of mice used for repeated measurements.

Organs	Uses	First		Second		Third		Fourth		Fifth		Sixth	
		Body (g)	Organ (g)	Body (g)	Organ (g)	Body (g)	Organ (g)	Body (g)	Organ (g)	Body (g)	Organ (g)	Body (g)	Organ (g)
Heart^a^	Data-out	26.2	0.158	26.9	0.156	24.5	0.159	24.3	0.164	24.1	0.154	23.1	0.158
	Data-in	26.7	0.159	26.7	0.157	25.9	0.162	23.8	0.164	23.2	0.160	23.0	0.161
Orbit^a^	Data-out	26.2		26.9		24.5		24.3					
	Data-in	26.7		26.7		25.9		23.8					
Thorax^b^	Data-out	23.9#		23.3#		20.1§		19.7§		19.3§			
	Data-in	23.7#		23.5#		20.1§		19.9§		19.2§			

^a^The organs were derived from 33-week-old male DBA/1 mice. These mice weighing 23.0–26.9 g were purchased from Janvier (Janvier Labs, France).

^b^The organs were derived from male C57 BL/6 J mice at the age of # 10 weeks and § 6 weeks. The animals were delivered from Charles River Laboratory (Sulzfeld, Germany).

The corresponding sectioning-guided models, based on the values of the target positions measured in data-out blocks, were constructed. We used the cutting planes adjusted according to the sectioning-guided models and started the cutting of the data-in blocks paired. The specimen holder's cutting-forward distance (expected) to hit the surface mark of targets on the data-in block was recorded (Tabs S4, S5, and S6) depending on the cutting strokes counted. The expected cutting-forward distance is often at variance with the actual cutting-forward distance to stroke each target center due to potential bias of specimen, operation, and measurement. To test the feasibility of the method, we recorded the actual central position (actual cutting-forward distance) of each target for comparison. A high similarity in the cutting-forward distances measured between the expected and actual values was found. Comparisons with Student paired T-tests (expected and actual) for each kind of organ resulted in no statistical significance (N = 8–18, for all, *p* > 0.0612–0.774). The overall coefficients of variation from 4 to 6 rounds tested at different times were rather small, ranging from 0.0290 to 0.0357 for the expected values or from 0.0237 to 0.1125 for the actual values. To test accuracy testing on cutting-forward distance/place of actual measurements and of the expected values in data-in tissue blocks, we pooled all the values collected in the three kinds of tissues and made comparisons of the values between expected and measured. The comparisons yielded a statistically significant correlation (see Fig. 3B). With the method, we were successful in securing the sections that contained the targets in their central or proximate positions (Fig. 4) in all the repeat experiments. The results indicate that the technique is also robust, and the accompanied tools for trimming and embedding are also reliable.

## Discussion

Laboratory daily work on histology contributes to the development of the technique when we managed to acquire a section that contains a whole intraorbital optic nerve lineament for morphological investigation of the optic nerve and the orbital contents^27,28^. Since the optic nerve is widely accepted as a landmark, the sections with the nerve included would be standardized for valid morphometric analysis and comparison. There are no existing methods available to meet the demand. Therefore, we first developed a two-time embedding method (see Methods and Fig. S5). The first embedding was performed traditionally, and the second embedding was performed in an oriented embedding fashion so that the following sectioning plane is parallel to the grossly running direction of the intraorbital optic nerve. In addition, calibrated trimming, and an embedding guide (Fig. S5) were used to minimize discrepancies in the subsequent sectioning among the samples. Having utilized calibrated trimming guides and the multiembedding technique, five orbital samples were objected to sectioning. As a result, we observed sections containing whole intraorbital optic nerve outlines in 3 orbital tissue blocks in our trial tests. We missed the whole nerve outlines containing sections in the remaining 2 samples. The failures are supposed to be due to a shortage of precise spatial positions of the target centers in the embedding blocks.

To localize the target centers (in this case, we took the endpoints of the nerve as two targets—optic nerve in the optic duct and optic nerve in the connection to the eyeball for the whole intraorbital nerve), we inset a positioning plate as a reference in the sample embedding block (Fig. S5). Under the spatial coordinate setting, the target precise positions (X, Y, Z) were determined secondary to serial sectioning in one embedding block (data-out) of orbital samples. According to these 3D data sets, a corresponding sectioning-guided model was created (Figs. 1 and S3), which helps to align the sectioning angle and to define the cutting-forward distance. We used the corrected cutting plane and the predetermined cutting-forward distance to cut the other four identical embedding samples (data-in), the sections containing the whole intraorbital optic nerve lineaments were easily obtained in all the embedding samples to be cut (100% success rate) in our trial tests. To test the reproducibility of the method, we used 8 more right orbits and paired them up into 4 pairs (Table 3). The results were still satisfactory (Fig. 4B) and the overall CVs are small during the repeat experiments in different periods (Tables S1–S4). In addition, the labor-intensive workload is greatly lightened.

To further validate the method, we produced the three-targeted models, in which the target spatial positions are known. Comparisons of the coordinate values at the target position between the foreknown and measured values with the method in different periods resulted in a high closeness and comparisons of the cutting-forwarding distance between the expected (predetermined with the method) and actual values also lead to a similar result. These results verified the reliability of the method.

Applications of this method in the hearts and thoraxes successfully yielded single sections containing three aortic valves or the entire thoracic trachea in their center positions (Fig. 4). With those outcomes (Fig. 4), (1) we have relatively unified target-contained sections for each kind of organ, which would facilitate efficient comparisons; (2) the grossly morphological structures with the targets included are obtained without obvious deformation, which would ensure correct measurement of morphometry; (3) local lesions with respect to the landmarks would be easily localized.

A successful application of the method depends on a close similarity between the data-out and the data-in embedding blocks of pairs in (1) animal background, (2) targeted specimen trimming, and (3) orientation embedding of the targeted specimen.

### Animal background

The close similarity in age and body/organ weights of a strain and sex, between the paired animals (e.g., data-out and data-in) is a prerequisite for the application of the method. In addition, identical histological processing (e.g., targeted organ dissection and fixation) is essential for both the data-out and data-in organs or tissues. In the present study, therefore, matched pairs of the animals were made depending on the closest similarity in the body (or related organ) weights and the ages of a sex and strain group (Table 3).

### Targeted sample trimming

Identical trimming before embedding is critical for the precision of sectioning. For proper specimen processing by trimming, general knowledge and a rough estimation of the target positions where cutting goes are necessary. To make an identical trimming, a calibrated tissue cutter for orbits and a trimming guide for the thoracic block were used to trim to the same size in the paired tissues. For the heart (aortic valves), a plaster mold (see Methods) specifically designed to form arrayed mouse heart-shaped cavities, a “receiving matrix” was prepared for identically orientational trimming. Inserting heart samples directly into the cavities allows the samples to be effortlessly positioned into a uniform and optimal orientation and trimmed to an identical size (Fig. S1). In the longitudinal sectioning of the thoracic tracheas, the backs were inclined in trimmings at an angle of 81 degrees (Fig. S4) so that right sections can be obtained if the sectioning face parallels the embedding block bottom.

### Orientation embedding

Embedding guides are specially designed for orbit, heart, and thoracic embedding to yield a unified orientational embedding block for each kind of organ. With the embedding guides, we effortlessly align and embed samples in a unified orientation. The embedding guides provide a simple way to prepare the orientational embedding in a rapid and standardized manner and thus reduce the time and effort of operators. As an example of the two-time embedding technique (Fig. S5), orbital samples are first embedded traditionally, and trimming and realignment of the embedding direction in the secondary embedding are performed so that the final embedding sectioning plane is well within the capacity of the cryostat. (Note: the capacity for the cryostat of current use is 15–20° of angles in all the directions of alignment of cutting).

Target-hit sectioning is facilitated by the use of sectioning-guided models that are designed from the coordinate data of the data-out block. The sectioning-guided model is intended to adjust the cutting plane and to determine the specimen holder cutting-forward distance for the data-in tissue block paired. The cutting plane alignment for tissue blocks is important not only for obtaining sections containing the entire target outlines but also for facilitating the histomorphometric evaluation and comparison and the diagnostic location and measurement of pathological conditions affected^29–34^. The sections with a divergent sectioning plane would lead to distortion of the targets in one direction and over-or under-estimation of values^35^. With the established cutting plane and specimen holder cutting-forward distance, a section containing those target tissues in their central aspect is acquired from the data-in block. Thereafter, any tissue blocks (e.g., from colonial animals) similar to the data-in block will be cut with the same cutting plane and cutting-forward distance, and quick and accurate target-containing sections will be easily acquired.

The trimming guides, the heart plaster mold for trimming, the embedding guides, and the two-time embedding technique, which adjoined the method, ensure identical histological processing, in a reproducible manner, minimize random errors, and thereby may contribute to the reduction of the labor, and the number of animals needed.

The method necessitates an additional preparation of a sectioning-guided model and the following alignment of the sectioning plane, the mean duration of which is approximately 20–30 min. The time of the creation of the sectioning-guided model and manual adjustment of the sectioning plane could be reduced if the adjustment of the cutting plane could be replaced by an automated procedure. It is possible to develop an algorithm calculating the proper cutting plane based on the coordinates of the centers of targets. In such a case, it would only be necessary to acquire three-dimensional positions of target centers in the embedding blocks and the program would determine the cutting plane and cutting-forward distance efficiently and accurately.

In conclusion, we developed a four-step method to efficiently cut a section containing the target centers in histological specimens. Tests on repeat experiments confirmed that the method is reliable. The overall coefficient of variation is very low. The adjoined tools—various tissue-specific formulated calibrated trimming and embedding guides, and an organ-shaped cavity plaster mold, and the two-time embedding technique, which were used in the experiments, are also proved to be reliable, bear great potential to become a common supplemental tool for histology, and may contribute to the reduction of the labor, and the number of animals needed.

## Limitations

The method has potentially wide use in studies of normal structures in native animals. Pathological examinations with this method can be performed on the localized focal lesions in substantial organs—brain, kidney, liver, bone, etc. Site-prone diseases, such as atherosclerosis in the bifurcation or curve of arteries can also be investigated with this method for efficient comparison. However, the morphological diversity of local lesions may limit the application of the method. In addition, there are obvious limitations borne in the technique, which should be considered before the application.

### The number of tissue blocks

Two embedding tissue blocks are at least needed for the present experimental setting. One tissue block provides the target spatial locations with ordinate data (X Y Z). According to those data and the subsequently built sectioning-guided model, the adjusted sectioning plane is used for cutting the other similar tissue block. The data on the target locations can be obtained by Micro-CT or in vivo MRI, an ultrasonic diagnostic imaging system, and PET measured in one animal, on the condition that the targets have enough resolution to be distinguished from the background. Therefore, in such a situation, the animal, which provides the target location data, will not be required. It should be acknowledged that in such a case, the imaging-guided sectioning plane in the same animal may have superiority over the present technique since the deviated data may exist between the two animals or tissue blocks.

### Tissue block size

In the present setting, the maximal volume (long, wide, and high) of the tissue block is better less than 25 × 25 × 25 mm^3^ due to the limitation of the chuck of the cryostat, the slide of glass used to hold the tissue and section, and the staining facilities in a routine laboratory. For larger tissue blocks, another cryostat (e.g., a sliding microtome), and larger custom-made slides and staining facilities can be used.

### Cutting plane angle (between the edge surface of the blade and the cutting face of the specimen block)

The capacity of the cryostat used is in the range of 0–20° at the cutting angle. A larger angle adjustment for the alignment of the sectioning plane is invalid. In such a case, initial adjustment with trimming or multi-embedding just as in the way orbits are treated is necessary so that the later alignment of the cutting plane falls within the capacity of the cryostat. A supplement of a wage or an inclined face cut in the tissue blocks will be used to increase the cutting plane angle when the tissue block is mounted on the chuck for cutting. However, note that this will increase the risk of cutting into the face of the chuck.

### Target size

The sections of 7 µm in thickness were cut out for the present experiments. Smaller targets or regions of interest may be missed during the histoprocessing. Thinner sections will compensate for the shortcoming by increasing the sensitivity. But the present setup has a minimum thickness of 5 µm for sectioning to secure a complete section.

## Methods

### Key custom-made tools

The following lists key custom-made tools. The tools were employed for unified trimming, the identical orientation of embedding, localization of targets, and target-hit sectioning. For more details concerning the preparation, refer to Supplementary Information.

#### Coordinate/checked paper

It was used for localization of targets in the sections from data-out embedding tissue blocks and definition of the desired sectioning plane.

#### Positioning plates

The plates were inset in tissue blocks, and they served as landmarks for the orientation of embedding and reference lines when determining the positions of the targets.

#### Heart plaster molds

They were custom-made heart holders and were used for the orientation of the samples during trimming.

#### Trimming guides

They included thoracic trimming guides and orbital trimming guides. The guides were specially designed for unified trimmings among the same kind of organs.

#### Foil embedding molds

They were aluminum-foil-made containers. The containers included round foil embedding molds and cuboid foil embedding molds. The formers were used for the embedding of orbits and hearts and three-targeted models, and the latter was used for the embedding of the thoraxes.

#### Embedding guides

They were a circle or straight lines and indicated positions for the corresponding samples, positioning plates, or embedding molds to be placed.

#### Three-targeted models

For validation of the protocol and setups, three targets of different colors in OCT embedding blocks were created (Fig. S2). The spatial positions of the three targets were known.

#### Sectioning-guided models

They were paraffin blocks with fine needles inserted on the upsides. The sectioning-guided models were used for adjustment of sectioning plane cut in paired data-in tissue blocks.

### Animals and specimen preparations

All animal experiments were performed in accordance with Directive 2010/63/EU and approved by the Government of Upper Bavaria, reference number: 55.2Vet-2532.Vet_02-17-94, based on prior evaluation of animal study plan design and group sizes by the certified bio-statistician Dr. Peter Klein. The description of all procedures involving animals was done according to the ARRIVE Guidelines in reporting in vivo experiments^36^.

The mice used on the strain, age, body weight, and source are summarized in Table 3. Two organs bearing a higher similarity in weight/size were paired for hearts. Right orbital or thoracic tissue was made in pairs according to a closer similarity in the corresponding sourced mouse body weight. One of the matched pairs serves as a data-out organ or data-out block after embedding, and the other one serves as a data-in organ or data-in block.

#### Heart (aortic valves) preparations

The hearts were excised from the euthanized animals. After removal of the fat tissues and pulmonary arteries, the samples were fixed in 4% paraformaldehyde at 4 °C temperature for 48 h. Subsequent trimmings (Fig. S1) were performed with a vertical crosscut on the heart at the position of 4 mm from the tip, with aid of the heart plaster mold and the cutting matrix. This process is repeated to make more identical trimmed hearts for each pair. The trimmed samples were kept in 30% (w/v) sucrose (prepared in PBS) at 4 °C until use.

#### Orbital preparations

The animals were euthanized, and a complete dissection of the orbital and periorbital areas was performed, as described previously^27,37^. In brief, the head was dissected, and the skin, the surrounding connective tissue, and the brain and teeth were removed, but all orbital tissues, eyelids, and adjacent tissues remained intact. After fixation and decalcification, the tissue blocks were trimmed with coronary cuttings at the positions of the anterior and posterior canthi. Median sagittal cutting with a tissue slicer (Tissue slicer, Cat# 51425, Stoelting, USA) divides the block into left and right orbits. We used the right orbits for the present study. The trimmed samples of 6 mm in length were kept in 30% (w/v) sucrose (prepared in PBS) at 4 °C until further processing.

#### Thoracic trunk preparations

The mouse in a supine position was snap-frozen, and an identical trimming was performed (Fig. S4) with a trimming guide. Since the thorax is too large for the capacity of the cutting machine, we trimmed the specimens to a size that can be compatible with the present cryostat capacity (specification for the maximum size—25 × 25 × 25 mm^3^) but kept the desired structures intact. Cares were taken to trim the back of the trunk in such a cutting plane that the angle between the cutting plane and the bottom (abdominal end side) was 81°. Perpendicular trimming to the horizontal plane was verified with the tissue slicer. The trimmed samples were preserved at − 20 °C until needed.

### Orientation embeddings

For the tissue orientation embedding of hearts and orbits, the trimmed tissue blocks were incubated in OTC for 5 min at room temperature after incubation in 30% (w/v) sucrose. The blotted samples or the frozen thoracic trunks and positioning plates were placed on the corresponding positions of the embedding guided slides, and the standing positions were fixed with freezing by moving the slides onto a supercold metal block or with the snap glue. A foil mold was sheathed onto the sample and corresponded to the circle in black and filled with OCT after. The frozen tissue blocks were broken off from the embedding guide slide and stored at − 80 °C. The special steps for various tissues are described in the following.

#### Orbit embedding

There were two embeddings for major adjustment of the cutting direction (Fig. S5). The first embedding was completed with a trimming guide and the second embedding ended with the use of an embedding guide.

#### Heart (aortic valves) embedding

The trimmed heart was placed on the indicated position of the embedding guide slide, with the joint between the right and left ventricle anterior walls corresponding to the black dot of the embedding guide (Fig. S6). A positioning plate was erected in the demarcated position.

#### Thoracic trunk embedding

For the identically oriented embedding, the specimen and a rectangular foil embedding mold were placed on the corresponding positions of the embedding guide without a positioning plate inset (Fig. S4). This is because the bottom face (the abdominal side of a trimmed sample) is complete and continuous (rectangle in shape), and the edge (level of the fifth rib) itself in each section can serve as the X-axis (the line between superior edges of both sides of the fifth rib) and the perpendicular line through the left end of the axis on the 2D section after cutting is a virtual Y-axis.

### Sectioning and collection

The foil aluminum film was stripped of the tissue block, an identical shape was trimmed between the pairs, the upside of the tissue block was secured on the specimen disc with OCT (the bottom of the embedding block was cut first), and the disc was mounted on the specimen holder of a cryostat. The cutting direction was adjusted so that the cutting plane was parallel to the bottom of the data-out embedding blocks. The cryostat (Cryostat CM1850, Leica Biosystems, Germany) was set to 7 µm at section thickness and − 20 °C at chamber temperature. The tissue block was balanced in the chamber for at least 45 min before starting sectioning. A serial section of 7 µm in thickness with an interval of 100 µm was conducted as we did before^27,28,37,38^.

To ensure intact sections were collected at any desired places, the adhesive tape-aided (Adhesive tape, Cat# 57405, Tesa, Germany) sectioning technique was used^28^. Briefly, a slip of the adhesive tape was clipped, held with fine forceps at a corner, and pressed with the adhesive side of the tape onto the trimmed cutting surface. For best adhesion results, light pressure was applied to the tape with soft tissue. The sample was sectioned slowly, evenly, and continuously without the use of a brush or anti-roll device. Forceps were used to pick up the freshly sectioned tissue by the corner of the tape and adhere it to the double-adhesive type on a slide.

For sectioning of the data-in tissue blocks, (1) the sectioning plane was aligned according to the sectioning-guided model, (2) the data-in block was mounted on the cryostat with the same orientation as the sectioning-guided model, and (3) the target 2D positions (x, y) were marked on the cutting face in reference to the positioning plate (Fig. S3B). Start cutting and record the cutting-forward distance (expected position) to hit the first labeling position on the embedding guide.

Note that (1) for the alignment of the sectioning plane, mount the sectioning-guided model in the cryostat and forward or reverse the specimen head, adjust the cutting plane angle and revolve the disc so that the blade hits all the tips of needles concurrently at one stroke of cutting; (2) for registration of the sectioning-guided model in the specimen disc holder, the sectioning plane or angle was fixed by tightening the screw and the orientation position of the positioning plate was traced with straight lines on the specimen head front both to the left and right of the disc; (3) for the same positioning as the sectioning-guided model, a paired data-in block replace the model and orientate the data-in block (with the positioning plate parallel to the traced lines on the head surface) with revolving the disc (Fig. S3); (4) for the labeling the 2D (X, Y) central positions of the targets on the initial cutting plane (bottom of the data-in embedding block), the embedding guided paper printed in mirror style was clipped off along the demarcation of foil embedding mold and pasted on the bottom with a domestic glue (the positioning plate in the embedding block was lined up with the corresponding line of the embedding guide); (5) for location of the target 2D positions on the embedding guide, mark the places with colors.

With the cutting plane aligned, cutting was performed first with trimming by a 20 µm thick section, until 1000 µm before the expected position was reached (Fig. S7). Then, the section was cut into 7 µm sections, and each section was inspected for the target structures in the tissue block or colors for the three-target molds. The position (actual cutting-forward distance) was recorded when it or when they were present in the section. The inspection was carried out with a hand magnifier examination on the cutting face. If the targets are present on the cutting surface, change the blade with a new one and collect the section at once. Be sure that the cutting face angle does not change before finishing cutting the block. The collected sections were kept at − 80 °C.

### HE (Hematoxylin and Eosin) staining

The sections were thawed at room temperature for 30 min before the procedures of HE staining were performed. The procedures comprised mainly fixation of 4% paraformaldehyde, 3 min Harri’s hematoxylin staining (Harri’s hematoxylin, Cat# HHS80, Sigma), and 40 second eosin staining, and 20% glycerin-aided (Glycerin, Cat# 3783.1, Carl Roth, Germany) coverslipping. Staining was applied only in the sections of the tissue blocks (the staining was avoided in the sections of three-targeted models since the targets had been labeled with various colors). Traditional HE staining protocol was adopted for the slides indicated in Table S7, Supplementary Information.

### Imaging and acquisition

Macro examinations were performed on the tissue staining and three-targeted model sections under an epi-microscope (epi-microscope, Cat# NH99.1, Carl Roth, Germany), and the images were acquired with a Canon digital camera (Canon camera—EOS 600D, China) and recorded with 3456 × 2304 pixel resolution. As a reference, a piece of coordinate/checked paper was photographed under identical light conditions except that the focus was adjusted. These conditions include shooting distance (18 cm), aperture values (1/18), light intensive correction (+ 2), manual focus, av mode, and background (black for the three-targeted models and white for the rest).

### Determination of coordinate values of target positions

The JPEG images of the coordinate/checked paper and the section of data-out blocks were opened with Photoshop (Photoshop Element 6, Adobe). First, select and copy the whole image outline of the section and then paste or overlay on the grid square of a coordinate (Fig. 2A1/2). The light transparency degree (~ 50%) was adjusted, and a merged image of the pure red coordinate and target structure was shown (refer to Fig. 2A3). Align the image of the section so that the borderline of the positioning plate is moved in correspondence with the line of coordinate. Since we reverted the tissue block and cut the bottom face first, we accordingly flip the image in mirror-style when analyzing the coordinate values (Fig. 2A3).In reference to the X-axis, the initial point, and the virtual Y axis through the initial point of the positioning plate, each target 2D position (X, Y) was determined (Fig. 2A4/5). Only the position of the target central point for each target was recorded in one section. The value of Z indicates the cutting-forward distance of the specimen holder. The coordinate values were determined under a space rectangular coordinate setting in Fig. 1. The average value of each axis value (X, Y, Z) was regarded as the center point of each target in the embedding block. In orbital sections, there are two targets to be localized for the whole intraorbital optic nerve. They are both ends of the intraorbital optic nerve—the optic nerve in the duct and the connection with the eyeball. Left, right, and posterior valves are the targets in the sections of the heart. The middle points in the ascending aortic wall between the jointing dots of the corresponding valve are defined as the target central points of the valves for each section. In the thorax, the central point in the trachea at the level of the first rib of both sides is supposed to be the first target. The second target is the central point of the bifurcation in the trachea.

### Data analysis

Data are presented as means ± SEM. The software, SPSS (SPSS-v.11, IBM Corp USA) was employed for analysis of correlation, paired or multiple comparisons of means. A *p* < 0.05 is considered statistical significance.

## Supplementary Information

Supplementary Information.
