# Supplementary material for: Definition of a sectioning plane and place for a section containing hoped-for regions using a spare counterpart specimen
Source: Sci Rep. 2022 Aug 3;12:13342. doi: 10.1038/s41598-022-17380-z (PMC9349253; doi:10.1038/s41598-022-17380-z)
Supplement: Supplementary file 1 — Supplementary Information. [file 41598_2022_17380_MOESM1_ESM.docx]

**Supplementary Information**

Definition of a sectioning plane and place for a section containing hoped-for regions using a spare counterpart specimen

Zhongmin Li^1*^, Goetz Muench^1^, Clara Wenhart^1^, Silvia Goebel^1^ & Andreas Reimann^1^

^1^Advancecor GmbH, 82152 Martinsried, Germany.

***Corresponding author**

Zhongmin Li, Advancecor GmbH, Lochhamerstr. 29 A, 82152 Martinsried, Germany.

Tel: +49(0)89-2000204-13 Fax: +49(0)89-2000204-19 E-mail: [li@advancecor.com](mailto:li@advancecor.com)

1. **Supplemental Materials and Methods**

**Preparations of custom-made tools**

**Coordinate/checked paper:**It is made with PowerPoint (Microsoft Office, v8) and printed out in the finest absolute red line and high quality. The minimum square was 1 × 1 mm² and was confirmed by a Vernier caliper (Cat# 201023, Vernier caliper, Vogel, Germany**)**.

**Positioning plates:**The plates are 10 x 10 x 2 mm³ in size. They are used in the preparation of three-targeted models and orbit or heart embedding and are made of fresh Bavaria carrot in Germany. The chips were produced by cuttings in a cutting matrix (Cat # TM-2000, Cutting matrix, ASI, USA). They serve as landmarks for the orientation of embedding and as X-axes (borderline close to the tissue or targets) when determining the positions of the targets. The initial points of the X-axis are the left end of the plates. On the horizontal plane, the line perpendicular to the X-axis and passing the initial point is regarded as the virtual Y-axis. For the preparation of embedding, the plates were fixed in 4% paraformaldehyde at 4 °C for 30 min and blotted semidry before use.

**Heart plaster molds:**They are custom-made sample holders (Fig. S1), which are used to align the samples during trimming. The Heart plaster mold efficiently creates a “sample receiving mold” with heart-shaped cavities that securely hold the hearts to ensure a unified orientation for trimming. The mold is constructed by insertion of a frozen heart into a pad of plaster (Plaster, Cat# B6756 PD, Amazon, Play-doh, China), with the front face of the heart down and ascending aorta parallel to the pad surface. For the preparation of a cast of the heart, the fat tissue, pulmonary artery, and right and left auricles were removed. For a unified trimming between hearts in pair, one heart in a pair was first to snap-frozen in -80 °C balanced ethanol, then sealed airtight in a tube, and finally kept in -80 °C until use. The frozen hard heart served as a cast and produced two heart plaster molds with an identically heart-shaped cavity. One mold was used for trimming the melting heart from the frozen cast, and the other mold was used for trimming the fresh paired heart. To grasp the cast in an identical direction easily, the frozen heart was held in a position with the sample anterior aspect up and ascending aorta parallel to a slide surface, and the position was secured on the slide with a drop of OCT (Tissue-Tek O.C.T. Compound, Cat# 4583, Sakura) freezing on a supercold metal plate (Fig. S1A). The supercold metal plate had been frozen on dry ice beforehand. Then the slide was turned over with the heart down and held horizontally with the heart facing the plaster, and the sample is pressed onto the plaster until it contacted the padded bottom to make a heart-shaped cavity (Fig. S1B). The heart was gently removed from the plaster, and a sample receiving matrix with heart-shaped cavities was left. For the preparation of the plaster pad, a soft plaster ball of 8 mm in diameter was placed on the center of a slip of hard paper plate (6 x 10 x 12 mm) on a horizontal plane, and a slide was placed on the ball, and horizontally put down by force until an interval between the slide and the plate was up to 3.5 mm. The half heart-shaped cavities created in the sample receiving matrix simplified the arrangement of hearts by providing well-fitting and distinct “placeholders” for the samples.

**Trimming guides**: Two kinds of trimming guides are used. The first one is the thoracic trimming guide. It is specially designed for unified thoracic trimmings. The sample positions and the trimming line were demarcated on one slip of paper. The demarcated paper was pasted on the reverse side of a slide. The sample to be trimmed was placed on the slide and lined up with the lines on the ventral side and abdominal face (Fig. S4). The other kind of trimming guide is the orbital trimming guide, which is one slip of paper pasted on the reverse side of a slide. There are lines of two right angles demarcated on the guide (Fig. S5). It is used for the preliminary adjustment of the cutting plane after a sample is embedded and trimmed on the slide. Thus the orbital trimming guide is also called the initial orbital embedding guide.

**Foil embedding molds:**They are custom-made from aluminum foil (aluminum foil, Cat# 0954.1, Carl Roth, Germany) and described before^1^. Briefly, a strip of 15 mm wide rectangular aluminum foil was rolled onto a syringe shaft of the diameter required (we used a diameter of 17.3 mm) and fastened with a piece of adhesive tape. The cylinder roll was pulled down, and the foil embedding mold was ready for use. In the same way, a rectangle foil embedding mold of 25 x 22 x 15 mm in size was created by rolling a strip of 15 mm wide rectangular aluminum foil onto a rectangular prism of the matched parameters. Round foil embedding molds are used for the embedding of orbits and hearts and three-targeted models. Rectangle foil embedding molds are employed for the embedding of the thoraxes.

**Embedding guides:** There are two kinds of embedding guides. The first is a slide with a slip of coordinate/checked paper pasted on the underside (Figs S2, S5, and S6). It contains a circle with a diameter of 17.3 mm and a line of 10 mm in length demarcated on the paper. The circle indicates the position of the cylinder foil mold and the line of 10 mm that of the positioning plate for identically oriented orbit, heart, and three-targeted model embedding. The other kind of embedding guide is one slip of paper on which there are two rectangles demarcated (Fig. S4). The small one indicates the place of the trimmed thorax and the large one is that of a rectangular foil mold. The black line on one side of the small rectangle is supposed to be an X-axi**s**. The left end (the equivalent of the left edge of the left fifth rib) of the black line is the initial point of the X-axis. The demarcated guides allow for effortless alignment of an identical orientation of the thorax during embedding.

**Three-targeted models:** For validation of the protocol and setups, we create three targets in an OCT embedding block (Fig. S2). These three targets are 2x2x2 mm³ in size and are labeled red, green, and blue. To uphold the spatial positions of the targets, three quadrangular prisms are prepared and constructed with the cutting matrix. These prisms are made from fresh Bavaria carrot (Germany). They are 2x2x3, 2x2x4, and 2x2x5 mm³ in size. For a unified orientation of embedding, the position of the positioning plate, three targets’ 2D positions (X, Y) – red (2, -1), green (8, -3), and blue (5, -7), and a foil embedding mold place are demarcated on a slip of coordinate/checked paper (Fig. S2) pasted on the reverse side of a slide. A positioning plate first stood straight upright on the corresponding positions on the slide, and then the three semidry prisms – 2x2x3, 2x2x4, and 2x2x5 mm³ – were erected upright on the marked positions – red, green, and blue, respectively (Fig. S2A). Snap glue (Snap glue, Cat# 621603, UHU GmbH & Co KG, Germany) was used to secure the positions in situ. The prisms were capped with the labeled targets by connections between a semidry red labeling target and a 2x2x3 mm³ prism, a green labeling target and a 2x2x4 mm³ prism, and a blue labeling target and a 2x2x5 mm³ prism (Fig. S2B). For the capping, the blocks of color targets were adjusted to line up and tightly connected to the upside of the prism with the aid of snap glue and a pair of fine tweezers under an epimicroscope. Once the prisms and the positioning plate were secured (20 seconds) in their places, the slide was transferred onto the super cool bar of the cryostat, and the foil mold was sheathed onto the targets and positioning plate. Care was taken to be sure the foil mold was lined up with the circle demarcated on the slide. First, drop the OCT into the foil mold to cover the bottom and then fill the mold up with OCT when the bottom OCT started to become white. The embedding bottom was cut first and supposed to be the original plane under the space rectangular coordinate setting (Fig. 1B1). The 3D central positions (X, Y, Z) for the targets are therefore (-2, -1, -4) for red, (-8, -3, -5) for green, and (-5, -7, -6) for blue. For validation of the experiment, the coordinate values of positions (X, Y, Z) for each target are randomly selected, but the values in extremities (e.g., above 10 mm) are avoided. The extremity values would lead to sectioning being unworkable since the cryostat has limitations on the embedding size, the cutting direction (angle), and so on. For labeling the targets, we first submersed mouse kidneys in 10% formalin overnight and then incubated them in 1% Eosin (eosin G, Cat# 7089.1, Carl Roth, Germany), 0.2% Fast green (fast green FCF, Cat# 0301.1, Carl Roth, Germany), and 0.5% Toluidine blue (toluidine blue O, Cat# 198161, Sigma) for 7 days for red-, green-, and blue- labeled kidneys. Various marked chips of 2x2x2 mm³ were produced with aid of the cutting matrix from the stained mouse kidneys. After washing in 30% sucrose, the semi-wet staining marked (draining with blot tissues) targets were ready for the capping and embedding.

**Sectioning-guided models:** They include 15-mm-high cylindrical (Ø17.3 mm) and cuboid (15x25x 22 mm³) paraffin blocks. The corresponding coordinate/checked paper of the embedding guides in horizontal mirror style was pasted on the surface. Fine needles (stainless steel minutien pins, Cat# 26002-20, Fine Science Tools, Germany) were inserted straight upright in the paper and paraffin at the position of the corresponding x and y values (Fig. S3). Straight lengths above the paper represent Z values in 3D (refer to Fig. 1). For example, the coordinate values of 3D central positions (X, Y, Z) for the targets are (-2, -1, -4) for red, (-8, -3, -5) for green, and (-5, -7, -6) for blue in the three-targeted model, and the values for the pin tips are thus (-2, -1, 8), (-8, -3, 7) and (-5, -7, 6) for corresponding red, green and blue, respectively, in the section-guided model (A3 of Fig. S3, also refer to Fig. 1). These operations were finished under the epi-microscope and upright needles inserted were confirmed with a right-angle ruler in all directions. The column sectioning-guided models are for sectioning of paired data-in blocks of orbits, hearts, and three-targeted models, and the cuboids’ sectioning-guided models are for sectioning of paired data-in tissue blocks of the thoracic trunk. The cutting plane, which slides the pin tips in one stroke, is regarded as the virtual sectioning plane (Fig. 1B).


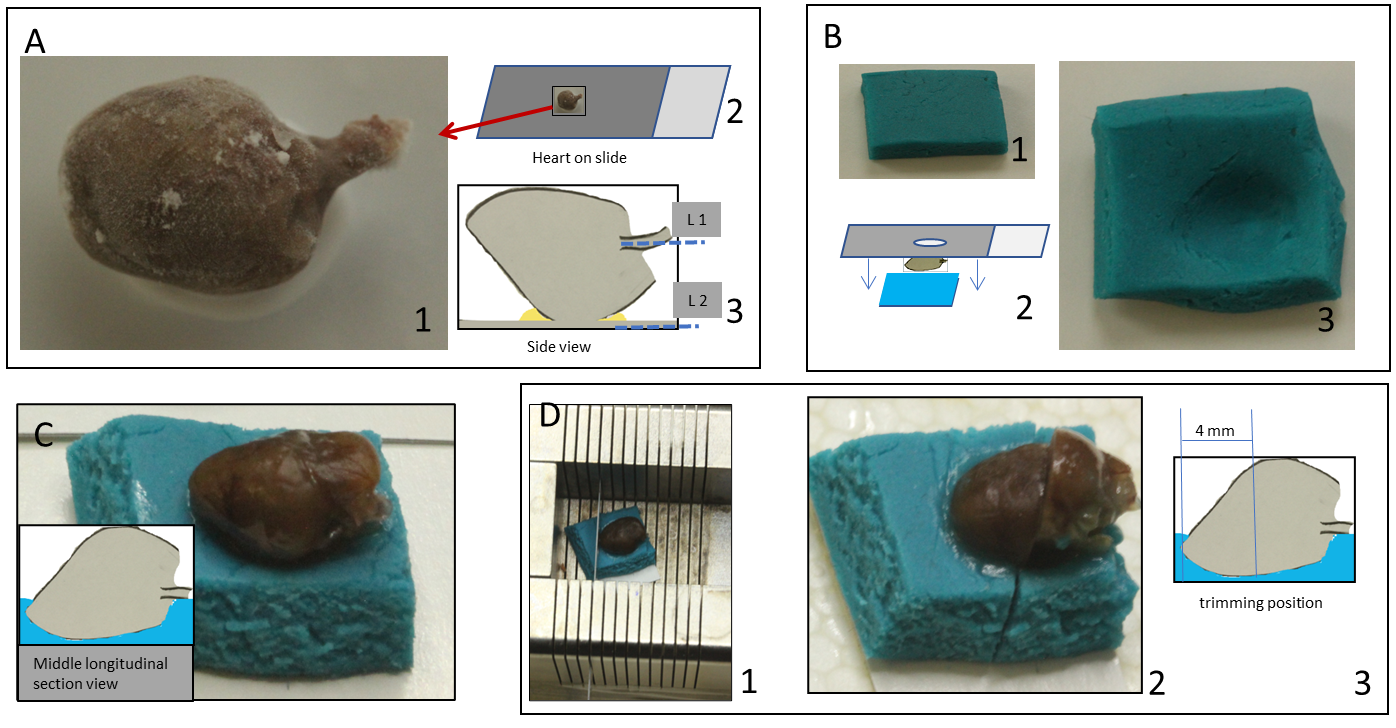


**Figure S1. Heart plaster mold and uniformed heart trimming.** A. The frozen heart is held in a position with the sample anterior aspect up and the ascending aorta parallel to a slide surface (L1 is parallel to L2 in A3) and the position is secured on the slide (A2) with a drop of OCT freezing on a supper cold metal plate. B. The cast (A2) is employed to insert into a pad of plaster (B1 and B2) to produce a sample receiving matrix with a heart-shaped cavity (B3). C. Another fresh heart paired is inserted into the matrix heart-shaped cavity. D. The heart is mounted onto the cutting matrix (D1). The trimming is completed after a cross-cutting perpendicular to the ascending aorta is made at the place of 4 mm from the heart apex (D2 and D3).

**Figure S2. Construction of three-targeted models.** A. A three-target embedding guide. The guide shows the corresponding positions of a foil mold (black circle), a positioning plate (blue line), and three targets (red, green, and blue dots) on a slip of coordinate paper pasted on the reverse side of a glass. The various colors (red, green, and blue) are labeled according to the values of the X and Y axes. B. The three different long prisms are placed on the corresponding positions and capped with the corresponding color labeling targets, and a positioning plate is stood firmly upright on the corresponding positions with aid of a snap-glue. C. A foil cylindrical mold is sheathed onto the prism targets and the positioning plate, and OCT mounting medium is filled into the mold and frozen for sectioning.

**Figure S3. Production of a sectioning-guided model, alignment of a sectioning plane, and determination of a cutting position (cutting-forward distance).**A. An identical paraffin block (A1) to the data-out and data-in tissue embedding blocks in size is used to reproduce the virtual sectioning plane with three needles. To localize the 2D (X Y) position of the targets on the initial (original) cutting surface, an embedding guide in a mirror style is pasted on one end of the paraffin block and marked (A2) as the corresponding positions in 2D (X Y). Fine needles are inserted in the paraffin at each marked position (A3) and kept upright. B. The sectioning-guided model is mounted on a chuck central portion with OCT or glue, and the chuck is put onto the chuck holder of a cryostat. The model orientation (direction) is tracked with lines labeled on the front face of the specimen holder (black lines in B1, which line up with the positioning plate) before the sectioning-guided model is replaced with a data-in block paired. The data-in block paired is mounted in the cryostat in the same orientation as that in the sectioning-guided model (B2). With the sectioning plane, cuttings are performed until the blade hits the one target position of 2D (X, Y) labeled on the surface of the tissue block (B3). The cutting-forward distance is recorded from start to hitting the first target 2D position on the date-in embedding block.

**Figure S4. Thoracic trunk dissecting, trimming, and embedding.** A mouse is fixed in a supine position on a foam plate with needles (A&E) and snap-frozen in the dry ice-balanced ethanol. After balancing at -20° C for 30 min, the upper edge of the sternum and the joint between both sides of the rib arch are labeled with black on the ventrally skinned body (A&E). The two cross cuts (reflected by the dotted lines in A and B) perpendicular to the horizon are performed through the marks mentioned respectively (see A and E). The front limbs are amputated along the trunk (A and E) and the upwarping zone in the joint of both side ribs is scappled (B and F). The trimmings are made in the separate block (F) of the thoracic trunk, with the reference to B and C. Cares are taken to trim them back (indicated by dotted lines in C1) of the trunk in such a cutting plane that the angle between the cutting plane through the lower border and the bottom is 81° (C and G). For the embedding, a black line inside the large black box in D is corresponding to the bottom face (abdominal side), the box formed with the black and blue lines to the specimen, and the bold black box to the embedding mold. At first, the specimen is put in the position demonstrated with the back down (H). Then a foil embedding mold is sheathed onto the tissue block and OCT is filled as in I.

**Figure S5. Orientated trimming and multi-embeddings of orbits.**Two referenced diagrams are drawn on the reverse sides of slides respectively as referred to A and B. A. There are two right angles displayed, colored red and blue respectively. For the first embedding, a right orbit is placed on the slide with the palate down and the medial and coronary boundaries corresponding to blue lines, as seen in C. After the sample is covered with OCT and frozen, two vertical cuttings along the red lines are made (D). With the underneath-cut side down, the first embedding issue block is put onto the circle in the other slide of an embedding guide indicated by B and E. For the second embedding, the positioning plate and the tissue block are aligned abreast to the left end of the blue line as seen in B and E. A foil mold is sheathed onto the block and filled with OCT as in F.

**Figure S6. Heart embedding.**A. A heart embedding guide. The guide shows demarcations on a slip of coordinate paper pasted on a slide. The black-lined circle indicates the position of a foil mold and the blue line the position of a positioning plate. The cutting face of a trimmed heart is put in the blue circle (B), with the joint in the anterior aspect between the left and right ventricles corresponding to the black dot. The left end of the blue line is virtually an initial point. A positioning plate is stood upright on the corresponding positions with aid of a snap-glue (B). C. A foil cylindrical embedding foil mold is sheathed onto the heart and the plate and moved to line up with the black-lined circle. The slide is shifted onto a super cool bar in a cryostat and OCT medium is filled into the mold. The tissue block is ready for sectioning after the medium is frozen.

**Figure S7. Sectioning flow chart.**


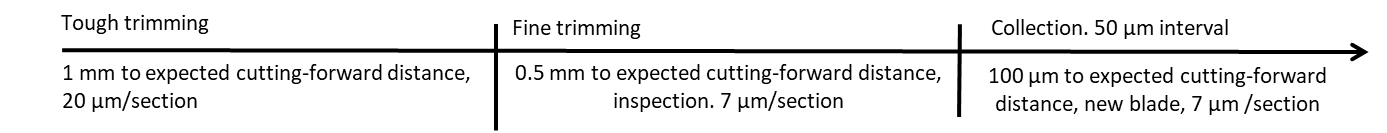


**Table S7. HE staining procedure.**

| 1. Fix in 4% PFA for 10 min. 2. Wash in deionized water. 3. Immerse in Harris hematoxylin for 3 minutes. 4. Wash in water. 5. Blue in running tap water for 10 minutes. 6. Immerse in eosin solution for 40 seconds. 7. Dehydrate sections with two 20-second washes in 100% ethanol. 8. Place in xylol for 30 seconds. 9. Mount in a mounting medium. |
| --- |

**2. Supplemental Results**

**Table S1. The coordinate values of the target central positions (ON duct, optic nerve in the duct; ON conn, optic nerve in the connection to eyeball) and the coefficients of variation (CV) from four runs of measurements in the orbital data-out blocks.**

| Targets | Positions | | Measured values (µm) | | | | |
| --- | --- | --- | --- | --- | --- | --- | --- |
|  |  |  | First | Second | Third | Fourth | CV |
| ON duct | X | | -2300.0 | -2300.0 | -2300.0 | -2100.0 | 0.04444 |
|  | Y | | -3600.0 | -3300.0 | -2850.0 | -2800.0 | 0.12163 |
|  | Z | | -5026.0 | -5250.0 | -5022.0 | -5120.0 | 0.02097 |
| ON conn | X | | -5300.0 | -4800.0 | -4400.0 | -4800.0 | 0.07638 |
|  | Y | | -5300.0 | -5000.0 | -5100.0 | -4800.0 | 0.04122 |
|  | Z | | -5426.0 | -5200.0 | -5424.0 | -5370.0 | 0.0199 |
| Total: | |  |  |  |  |  | 0.05409 |

Multiple comparisons among different rounds measured, with Tukey HSD, result in no statistical significance (N=24, for all, p=0.970857-1).

**Table S2. The coordinate values on the target central positions (left, right and posterior valves - L. val, R. val, P. val) and the coefficients of variation (CV) from six runs of measurements in the aortic roots of data-out blocks.**

| Targets | Positions | Measured (µm) | | | | | | |
| --- | --- | --- | --- | --- | --- | --- | --- | --- |
|  |  | First | Second | Third | Fourth | Fifth | Sixth | CV |
| L. val | X | -4300.0 | -4400.0 | -4300.0 | -4800.0 | -4100.0 | -4300.0 | 0.05354 |
|  | Y | -2400.0 | -2300.0 | -3200.0 | -2700.0 | -2800.0 | -2500.0 | 0.12344 |
|  | Z | -3150.0 | -3250.0 | -3220.0 | -3150.0 | -3200.0 | -3000.0 | 0.02796 |
| R. val | X | -5400.0 | -5300.0 | -5400.0 | -5700.0 | -5200.0 | -5500.0 | 0.0318 |
|  | Y | -2400.0 | -2400.0 | -2600.0 | -2700.0 | -2600.0 | -2800.0 | 0.06202 |
|  | Z | -3050.0 | -3250.0 | -3320.0 | -3150.0 | -3200.0 | -3050.0 | 0.03427 |
| P. val | X | -4900.0 | -4900.0 | -5300.0 | -5200.0 | -5000.0 | -5100.0 | 0.03223 |
|  | Y | -3100.0 | -2800.0 | -3700.0 | -3500.0 | -3200.0 | -3300.0 | 0.09616 |
|  | Z | -3150.0 | -3250.0 | -3220.0 | -3250.0 | -3200.0 | -3100.0 | 0.01865 |
| total: |  |  |  |  |  |  |  | 0.05334 |

Multiple comparisons among different rounds of measurements, with Tukey HSD, result in no statistical significance. (N=54, for all, p=0.994-1).

**Table S3. The coordinate values of the target centers (Trachea in., a trachea in initial; Trachea bi., a trachea in bifurcation) and the coefficients of variation (CV) from five runs of measurements in the data-out blocks of thoraxes.**

| Targets | Positions | Measured values | | | | | |
| --- | --- | --- | --- | --- | --- | --- | --- |
|  |  | First | Second | Third | Fourth | Fifth | CV |
| Trachea in. | X | -7300 | -7160 | -7100 | -6900 | -6750 | 0.0309 |
|  | Y | 9700 | 9800 | 9200 | 9600 | 9500 | 0.0241 |
|  | Z | -6390 | -6230 | -6180 | -5990 | -6090 | 0.0244 |
| Trachea bi. | X | -7000 | -7350 | -6800 | -6800 | -6900 | 0.0327 |
|  | Y | 6000 | 5800 | 5600 | 5980 | 6080 | 0.0327 |
|  | Z | -6390 | -6000 | -5870 | -6090 | -6290 | 0.0345 |
| total: |  |  |  |  |  |  | 0.0299 |

Multiple comparisons among different rounds measured, with Tukey HSD, result in no statistical significance. (N=30, for all, p=0.9925-1).

**Table S4. Repeatability testing on specimen holder cutting-forward distance (µm) measured in the sectioning-guided models (expected) and orbital data-in paired blocks (actual), and the coefficients of variation (CV) of four runs in different periods.**

| Targets | First | Second | Third | Fourth | CVact | CVexp |
| --- | --- | --- | --- | --- | --- | --- |
| ON duct | 5550[5674] | 5370[5210] | 5240[5460] | 5270[5370] | 0.0261 | 0.0357 |
| ON conn | 5432[5674] | 5418[5210] | 5670[5460] | 5320[5370] | 0.02722 |  |
| Total: |  |  |  |  | 0.02666 | 0.0357 |

Comparison of the values between expected and measured in data-in blocks, with Student paired T-test results in no statistical significance (N=8, P> 0.774 of 2-tailed). The numerals in brackets indicate the expected values and the numerals without brackets are those measured in the data-in blocks paired. ON duct, optic nerve in the duct; ON conn, optic nerve in the connection to eyeball.

**Table S5. Repeatability testing on specimen holder cutting-forward distance (µm) measured in the sectioning-guided models (expected) and data-in paired blocks (actual) of hearts, and the coefficients of variation (CV) from six rounds of different periods.**


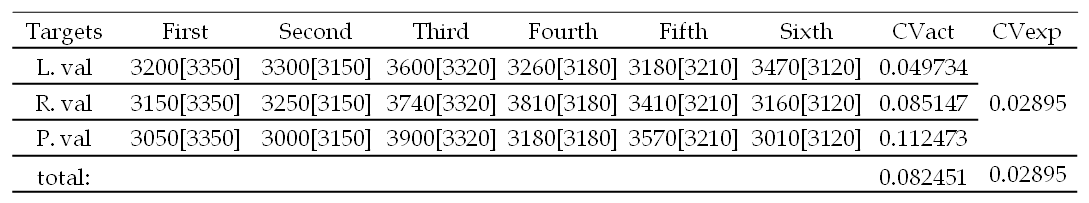
 Comparisons of the values between expected (in brackets) and measured in data-in blocks of one paired, with Student paired T-test result in no statistical significance (N=18, for all, p> 0.06492 of 2-tailed). L. val, left valve; R. val, right valve; P. val, posterior valve.

**Table S6. Repeatability testing on specimen holder cutting-forward distance (µm) of targets measured in the sectioning-guided models (expected) and data-in paired blocks (actual) of thoraxes, and the coefficients of variation (CV) from five rounds.**

**
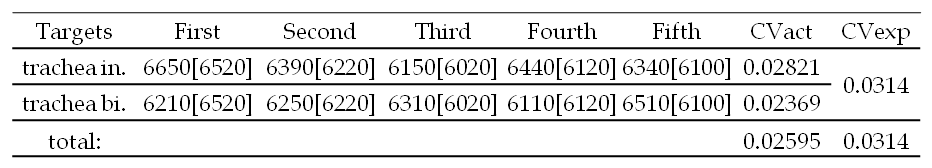
**Comparison of the values between expected (in brackets) and measured in the data-in blocks paired, with Student paired T-test results in no statistical significance (N=10, p> 0.0612 of 2-tailed). The numerals in brackets indicate the values expected and the numerals without brackets those measured in the data-in blocks paired. trachea in., trachea in initial; trachea bi., trachea in bifurcation.

1. **References**
2. [Li, Z., Ungerer, M., Faßbender, J., et al. Tissue block staining and domestic adhesive tape yield qualified integral sections of adult mouse orbits and eyeballs. PLoS ONE 16, e0255363, doi:10.1371/journal.pone.0255363](https://doi.org/10.1371/journal.pone.0255363) (2021).
